# Nudt21-mediated alternative polyadenylation of HMGA2 3′-UTR impairs stemness of human tendon stem cell

**DOI:** 10.18632/aging.103771

**Published:** 2020-09-26

**Authors:** Yangbai Sun, Hua Chen, Hui Ye, Wenqing Liang, Kun-kuan Lam, Biao Cheng, Yong Lu, Chaoyin Jiang

**Affiliations:** 1 Department of Musculoskeletal Oncology, Fudan University Shanghai Cancer Center, Department of Oncology, Shanghai Medical College, Fudan University, Shanghai 200032, China; 2 Department of Orthopedic Surgery, Shanghai Jiaotong University Affiliated Sixth People’s Hospital, Shanghai 200233, China; 3 Simmons Comprehensive Cancer Center, University of Texas Southwestern Medical Center, Dallas, TX 75390, USA; 4 Department of Orthopaedics, Shaoxing People’s Hospital, Shaoxing Hospital, Zhejiang University School of Medicine, Shaoxing 312000, Zhejiang, China; 5 Department of Orthopaedic Surgery and Sports Medicine, University Hospital of Macau University of Science and Technology, Macau 999078, China; 6 Department of Orthopedics, Shanghai Tenth People’s Hospital, Tongji University, School of Medicine, Shanghai 200072, China; 7 Department of Radiology, Rui Jin Hospital, Lu Wan Branch, School of Medicine, Shanghai Jiaotong University, Shanghai 200020, China; 8 Department of Orthopedic Surgery, Haikou Orthopedic and Diabetes Hospital of Shanghai Sixth People's Hospital, Hainan 570300, China

**Keywords:** HMGA2, tendon stem cells, stemness, pluripotency, Nudt21

## Abstract

Tendon-derived stem cells (TSCs) play a primary role in tendon physiology, pathology, as well as tendon repair and regeneration after injury. TSCs are often exposed to mechanical loading-related cellular stresses such as oxidative stress, resulting in loss of stemness and multipotent differentiation potential. Cytoprotective autophagy has previously been identified as an important mechanism to protect human TSCs (hTSCs) from oxidative stress induced impairments. In this study, we found that high-mobility AT-hook 2 (HMGA2) overexpression protects hTSCs against H_2_O_2_-induced loss of stemness through autophagy activation. Evidentially, H_2_O_2_ treatment increases the expression of Nudt21, a protein critical to polyadenylation site selection in alternative polyadenylation (APA) of mRNA transcripts. This leads to increased cleavage and polyadenylation of HMGA2 3′-UTR at the distal site, resulting in increased HMGA2 silencing by the microRNA *let-7* and reduced HMGA2 expression. In conclusion, Nudt21-regulated APA of HMGA2 3′-UTR and subsequent HMGA2 downregulation mediates oxidative stress induced hTSC impairments.

## INTRODUCTION

Tendinopathy accounts for about 30% of all musculoskeletal complaints in a general practice setting [1]. Both acute and chronic tendinopathy cause pain and morbidity, and may require long and expensive treatments including joint replacement [2]. Recent studies have revealed the presence of a stem cell population within tendons [3, 4]. These tendon-derived stem cells (TSCs) are capable of self-renewal and multipotent differentiation *in vitro* and *in vivo* [5]. TSCs play a primary role in tendon physiology, pathology, as well as tendon repair and regeneration after injury [6–8]. Since TSCs are often exposed to oxidative stress related to mechanical loading or injury [9], maintaining their stemness under cellular stress is critical for tendon tissue preservation and repair.

High-mobility group AT-hook 2 (HMGA2) belongs to the non-histone chromosomal high-mobility group (HMG) protein family. HGMA2 binds to DNA through three DNA-binding domains (AT-hooks) and modulates gene transcription by altering DNA structure and promoting the assembly of protein complexes in transcription factors. HMGA2 is a multifunctional protein involved in many cellular processes, and is best known for its regulatory role in stem cell self-renewal and differentiation along with oncogenesis [10–12]. HGMA2 is post-transcriptionally regulated by the *let-7* microRNA (miRNA) [13, 14]. The HMGA2 mRNA transcript contains seven complementary sites for *let-7* binding in its 3' untranslated region (3′-UTR) [15].

In eukaryotes, the 3′ ends of RNA transcripts are generated by site-specific endo-nucleolytic cleavage, followed by the addition of a poly (A) tail. Alternative polyadenylation (APA) leads to heterogeneous 3′-UTR lengths, rendering mRNA transcripts susceptible to different levels of negative regulation by miRNAs [16]. Interestingly, a recent study has shown that HMGA2 can avoid *let-7-*mediated silencing through APA-mediated 3′-UTR shortening [17]. Nudt21, also referred to as cleavage and polyadenylation specificity factor subunit 5 (CPSF5), is a subunit of the cleavage factor Im (CFIm) complex required for 3′-UTR cleavage and polyadenylation. Nudt21 is responsible for sequence-specific recognition of the element UGUA upstream of the poly (A) site (PAS), and thus plays a determinant role in APA [18, 19].

In this study, we investigated the function of HMGA2 in the maintenance of H_2_O_2_-treated human TSCs (hTSCs). Our data indicate that HMGA2 overexpression activates cytoprotective autophagy and protects hTSCs against oxidative stress-induced loss in stemness and multipotent differentiation potential. Oxidative stress increases Nudt21 expression, resulting in distal PAS selection and generation of HMGA2 transcripts with the long 3′-UTR. This in turn leads to increased *let-7*-mediated silencing and reduction of HMGA2 expression. Therefore, we identified Nudt21-mediated APA of HMGA2 3′-UTR as a novel mechanism that regulates HMGA2 expression and the stemness of TSC under oxidative stress, and thus plays a critical role in tendon homeostasis and regeneration.

## RESULTS

### HMGA2 overexpression protects hTSCs from H_2_O_2_-induced loss of stemness

**Figure 1:**
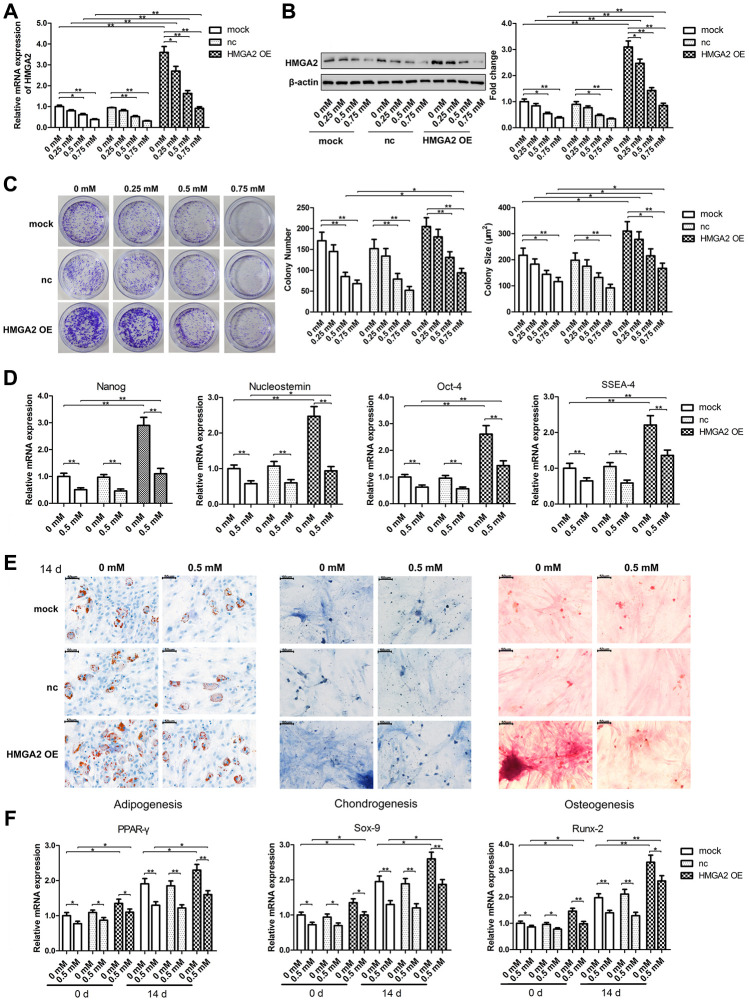
**HMGA2 overexpression protects hTSCs from H_2_O_2_-induced loss of self-renewal capacity and pluripotency.** hTSCs transfected with HMGA2 overexpression lentivirus (HMGA2 OE) or the negative control (nc) were subjected to H_2_O_2_ treatment at indicated concentrations for 24 h. Non-transfected cells (mock) were included for comparison. (**A**, **B**) The HMGA2 mRNA (a) and protein (b) levels were determined by qRT-PCR and western blot analysis, respectively. (**C**) Clonogenicity was evaluated by the colony formation assay. (**D**) The quantitative expression of stemness markers Nanog, Nucleostemin, Oct-4, and SSEA-4 of treated hTSCs were measured by qRT-PCR. (**E**) The multilineage differentiation capability was assessed as described in materials and methods. Scale bar = 50 μm. (**F**) The expression of markers for lineage-specific differentiation (PPARγ for adipogenesis, Runx-2 for osteogenesis and Sox-9 for chondrogenesis) at day 0 and day 14 of differentiation was evaluated by qRT-PCR. The data shown are from three replicates and are presented as mean ± SD. *p < 0.05, **p < 0.01.

**Figure 2:**
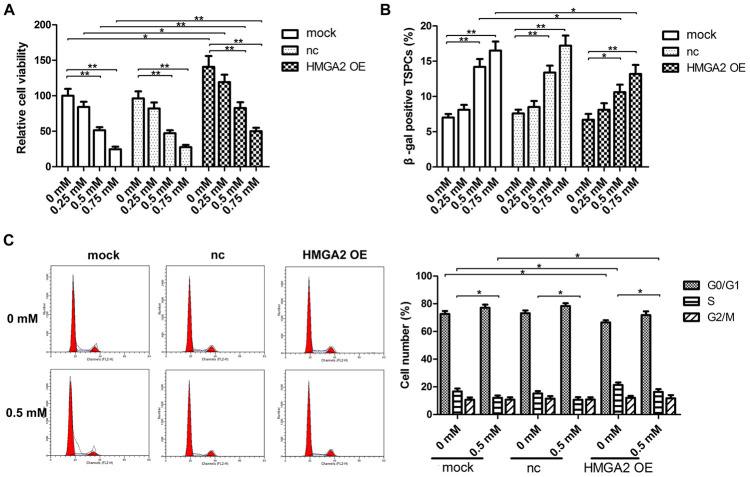
**HMGA2 overexpression protects hTSCs from H_2_O_2_-induced cell death, cell senescence and decreased S-phase cell number.** hTSCs transfected with lenti-HMGA2 (HMGA2 OE) or the negative control (nc) were subjected to H_2_O_2_ treatment at indicated concentrations. Non-transfected cells (mock) were included for comparison. (**A**) Cell viability was determined by the CCK-8 assay after 24 h of H_2_O_2_ treatment. (**B**) Cell senescence was assessed by β-gal staining after 72 h of H_2_O_2_ treatment. (**C**) Cell cycle analysis was performed by flow cytometry after 24 h of H_2_O_2_ treatment. The data shown are from three replicates and are presented as mean ± SD. *p < 0.05, **p < 0.01.

To evaluate the stemness of hTSC, we determined hTSC’s potential regenerative capacity using the colony formation assay. We also assessed the capabilities of hTSCs to differentiate into adipogenic, osteogenic, or chondrogenic lineage cells by monitoring the expression of lineage-specific markers in induced cells, along with the expression of stemness marker genes, such as Nanog, Nucleostemin, Oct-4, and SSEA-4. Similar to our previous findings [20], hTSCs exhibited reduced clonogenicity, decreased gene expression of stemness markers and impaired multilineage differentiation capability after H_2_O_2_ treatment (Figure 1C–1F). In this work, we found that H_2_O_2_ exposure also led to decreased mRNA and protein expression of HMGA2 (Figure 1A, 1B), a key regulator of stem cell stemness including promoting cell proliferation and maintaining multipotentiality [11, 21, 22]. HMGA2 overexpression restored potential regenerative powers as well as pluripotency of hTSCs impaired by H_2_O_2_ (Figure 1C–1F). Moreover, H_2_O_2_-treated hTSCs displayed reduced cell viability along with increased cell senescence and decreased number of S-phase cells, and these H_2_O_2_-induced cell impairments were prevented by HMGA2 overexpression (Figure 2A–2C).

### HMGA2 overexpression protects hTSCs from H_2_O_2_-induced impairments by activating autophagy

**Figure 3:**
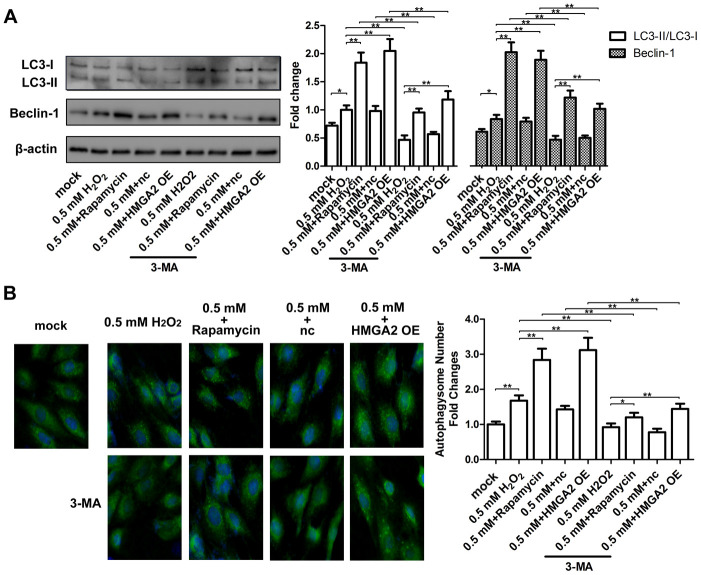
**HMGA2 overexpression activates autophagy in H_2_O_2_-treated hTSCs.** (**A**) hTSCs were incubated with rapamycin and 3-MA for 24 h, alone or in combination as indicated, and subsequently exposed to 0.5 mM H_2_O_2_ for 24 h. hTSCs were transfected with lenti-HMGA2 (HMGA2 OE) or the negative control (nc) in the presence or absence of 3-MA for 24 h, and subsequently exposed to 0.5 mM H_2_O_2_ for 24 h. Normal cells (mock) were included for comparison. The protein levels of the autophagic markers LC3-I, LC3-II, and Beclin-1 were determined by western blot analysis. (**B**) GFP-LC3-transfected hTSCs were subjected to treatments as described in a. GFP-LC3-labeled vacuoles (puncta) were detected by fluorescence imaging at 60× magnification. The data shown are from three replicates and are presented as mean ± SD. *p < 0.05, **p < 0.01.

**Figure 4:**
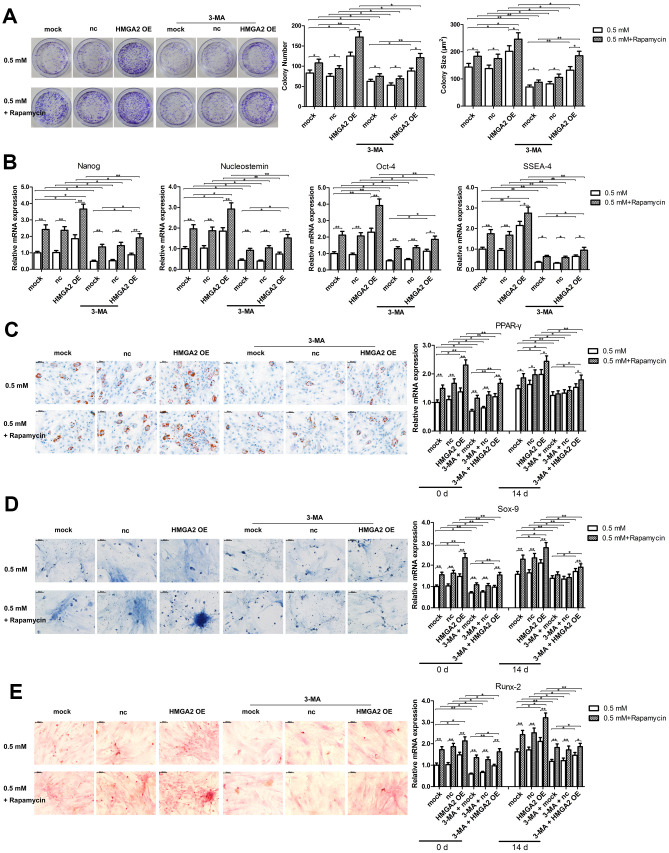
**Autophagy blockage abolishes the protective effects of HMGA2 overexpression against H_2_O_2_-induced loss of potential regenerative power and pluripotency.** hTSCs transfected with lenti-HMGA2 (HMGA2 OE) or the empty virus (nc) were incubated with 3-MA and rapamycin for 24 h, alone or in combination as indicated, and subsequently exposed to 0.5 mM H_2_O_2_ for 24 h. Untransfected cells (mock) were included for comparison. (**A**) Clonogenicity was assessed by the colony formation assay. (**B**) The quantitative expression of stemness markers Nanog, Nucleostemin, Oct-4, and SSEA-4 were measured by qRT-PCR. (**C**–**E**) The multilineage differentiation capability and the expression of markers for lineage-specific differentiation (PPARγ for adipogenesis, Runx-2 for osteogenesis and Sox-9 for chondrogenesis) was evaluated by qRT-PCR. Scale bar = 50 μm. The data shown are from three replicates and are indicated as mean ± SD. *p < 0.05, **p < 0.01.

**Figure 5:**
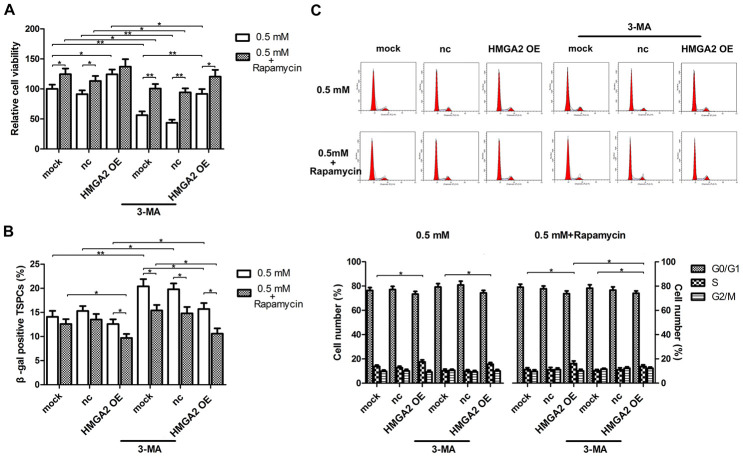
**Autophagy blockage abolishes the protective effects of HMGA2 overexpression against H_2_O_2_-induced cell death, cell senescence and decreased S-phase cell number.** hTSCs transfected with lenti-HMGA2 (HMGA2 OE) or the empty virus (nc) were incubated with 3-MA and rapamycin for 24 h, alone or in combination as indicated, and subsequently exposed to 0.5 mM H_2_O_2_ treatment. Untransfected cells (mock) were included for comparison. (**A**) Cell viability was determined by the CCK-8 assay after 24 h of H_2_O_2_ treatment. (**B**) Cell senescence was assessed by β-gal staining after 72 h of H_2_O_2_ treatment. (**C**) Cell cycle analysis was performed by flow cytometry after 24 h of H_2_O_2_ treatment. The data shown are from three replicates and are indicated as mean ± SD. *p < 0.05, **p < 0.01.

In our previous study, we showed that rapamycin activates cytoprotective autophagy in hTSCs, and thereby, protects against H_2_O_2_-induced loss of self-renewal capacity and stemness [20]. To investigate the mechanisms underlying the protective effects of HMGA2 on hTSCs, we assessed autophagy-related activities including LC3 cleavage and Beclin-1 expression by western blot analysis. Further, we also assessed the formation of GFP-LC3-labeled autophagosomes by fluorescence imaging. Similar to our previous findings [20], rapamycin activated autophagy in H_2_O_2_-treated hTSCs as indicated by increased LC3 cleavage, Beclin-1 expression and autophagosome formation. However, these autophagy-related changes induced by rapamycin were inhibited by the autophagy inhibitor 3-methyladenine (3-MA) (Figure 3A, 3B). HMGA2 overexpression induced similar autophagy-related changes in H_2_O_2_-treated hTSCs to rapamycin, and these changes were attenuated by 3-MA (Figure 3A, 3B). Moreover, results from multiple assays indicated that autophagy inhibition by 3-MA abolished the protective effects of HMGA2 overexpression on H_2_O_2_-treated hTSCs (Figures 4A–4E and 5A–5C). Thus, similar to rapamycin, HMGA2 overexpression protects hTSCs against H_2_O_2_-induced impairments through activation of autophagy. Interestingly, HMGA2 overexpression and rapamycin seemed to work additively to protect hTSCs from H_2_O_2_-induced damages (Figures 4A–4E and 5A–5C), suggesting that HMGA2 activates autophagy through a different pathway than rapamycin.

### Nudt21 is induced by H_2_O_2_ and down-regulates HMGA2 in hTSCs

**Figure 6:**
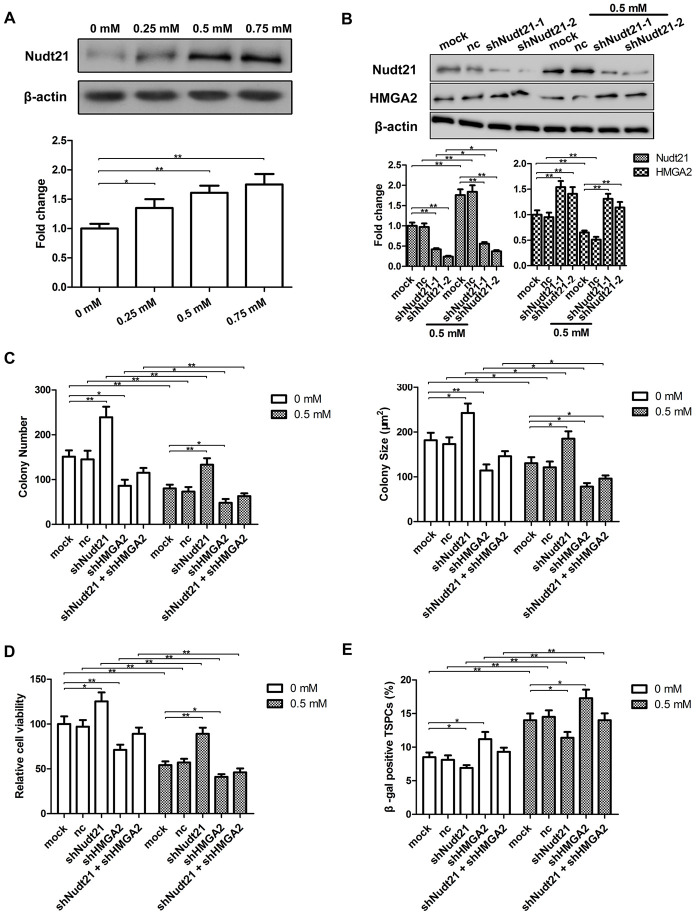
**Nudt21 is induced by H_2_O_2_ and down-regulates HMGA2 in hTSCs.** (**A**) hTSCs were subjected to H_2_O_2_ treatment at indicated concentrations for 24 h. The protein levels of Nudt21 were determined by western blot analysis. n = 3; *p < 0.05, **p < 0.01 vs. 0 mM H_2_O_2_. (**B**) hTSCs transfected with shNudt21-1, shNudt21-2 or a scrambled control shRNA (nc) lentivirus were cultured in the presence or absence of 0.5 mM H_2_O_2_ for 24 h. Nudt21 and HMGA2 protein levels were determined by western blot analysis. Untransfected cells (mock) were included for comparison. (**C**–**E**) hTSCs transfected with a scrambled control shRNA (nc), shNudt21-2 and shHMGA2, alone or in combination as indicated were cultured in the presence or absence of 0.5 mM H_2_O_2_ for 24 h. (**C**) Clonogenicity was evaluated by the colony formation assay. (**D**) Cell viability was determined by the CCK-8 assay. (**E**) Cell senescence was assessed by β-gal staining 72 h after H_2_O_2_ treatment. The data shown are from three replicates and are indicated as mean ± SD. *p < 0.05, **p < 0.01.

The results presented above have demonstrated that HMGA2 plays a critical role on hTSC maintenance under increased oxidative stress, and therefore, the loss of stemness and pluripotency in H_2_O_2_-treated hTSCs resulted from decreased HMGA2 expression in these cells. Previous studies have shown that HMGA2 can avoid downregulation by *let-7* through APA-mediated 3′-UTR shortening [17]. To identify whether APA involved in the loss of HMGA2 expression in H_2_O_2_-treated hTSCs, we examined the expression of Nudt21, a subunit of the CFIm complex responsible for PAS selective in APA [18, 19]. In contrast to HMGA2, Nudt21 expression increased in response to H_2_O_2_ stimulation (Figure 6A). In both H_2_O_2_-treated and untreated hTSCs, Nudt21 knockdown led to increased HMGA2 expression (Figure 6B and Supplementary Figure 1), indicating negative regulation of HMGA2 by Nudt21. In line with the function of HMGA2 in hTSC maintenance, HMGA2 knockdown led to decreased stemness and cell viability, as indicated by the colony formation, the Cell Counting Kit-8 (CCK-8) (Figure 6C, 6D). Further, we also observed an increase in cell senescence in both H_2_O_2_-treated and untreated hTSCs by the β-galactosidase (β-gal) staining assays (Figure 6E). Nudt21 knockdown showed opposite effects on both H_2_O_2_-treated and untreated hTSCs to HMGA2 knockdown (Figure 6C–6E), presumably mediated by increased HMGA2 expression secondary to Nudt21 knockdown. Moreover, the beneficial effects of Nudt21 knockdown were inhibited by HMGA2 knockdown (Figure 6C–6E). These results indicated that HMGA2 is negatively regulated by Nudt21.

### Nudt21 increases *let-7*-mediated silencing of HMGA2 through APA

**Figure 7:**
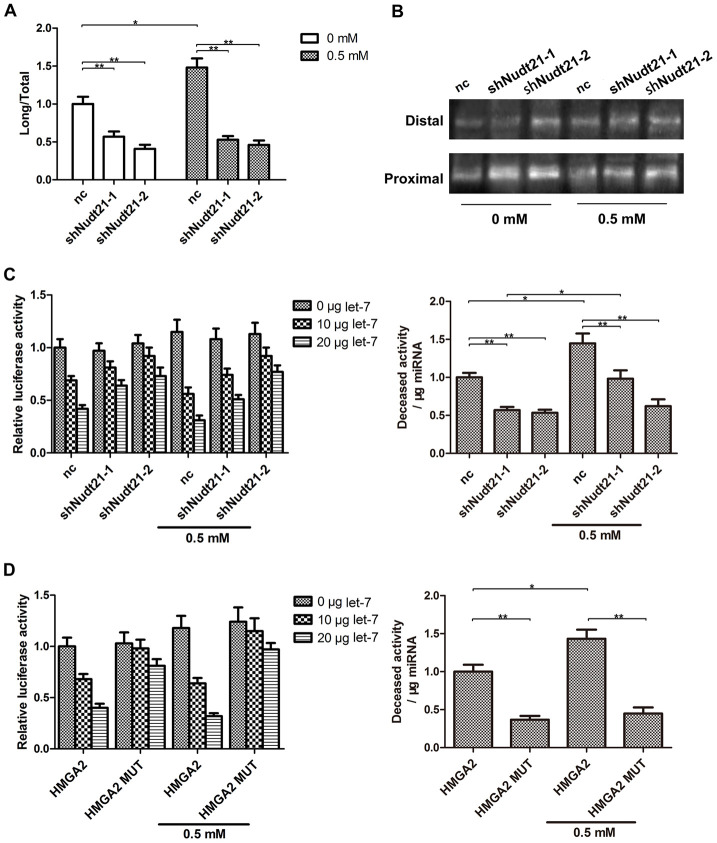
**Nudt21 upregulates *let-7*-mediated HMGA2 silencing through APA.** (**A**, **B**) hTSCs transfected with shNudt21-1, shNudt21-2 or a scrambled control shRNA (nc) lentivirus were cultured in the presence or absence of 0.5 mM H_2_O_2_ for 24 h. (**A**) The short and long HMGA2 3′-UTR transcripts were detected by RT-PCR. The long transcript-to-total transcript ratios are shown. (**B**) The short (Proximal) and long (Distal) HMGA2 3′-UTR transcripts were detected by 3′-RACE. (**C**) hTSCs were transfected with 100 ng psiCHECK2-HMGA2 along with shNudt21-1, shNudt21-2 or a scrambled control shRNA (control) and 0, 10 or 20 μg *let-7* as indicated in the presence or absence of 0.5 mM H_2_O_2_. The luciferase activity was determined 48 h after transfection. (**D**) hTSCs were transfected with 100 ng psiCHECK2-HMGA2 or psiCHECK2-HMGA2 MUT along with 0, 10 or 20 μg *let-7* as indicated in the presence or absence of 0.5 mM H_2_O_2_. The luciferase activity was determined 48 h after transfection. The data shown are from three replicates and are indicated as mean ± SD. *p < 0.05, **p < 0.01.

By binding to two UGUA elements upstream of the PAS, Nudt21 prevents CPSF-mediated pre-mRNA cleavage [19, 23]. Nudt21 knockdown has been shown to induce proximal PAS selection in several genes [24, 25]. The full length HMGA2 3′-UTR contains a proximal PAS localized between the first and second *let-7* binding sites and a distal PAS downstream of the seventh *let-7* binding site. To find out whether Nudt21 decreases HMGA2 expression through APA, we examined the transcript levels of the long and short HMGA2 3′-UTRs by RT-PCR and 3′-RACE analysis. Compared with control, hTSCs with Nudt21-knockdown exhibited higher levels of the short HMGA2 3′-UTR, a result of proximal PAS selection, with or without H_2_O_2_ treatment (Figure 7A, 7B). In line with this, Nudt21 knockdown enabled HMGA2 3′-UTR to evade *let-7*-mediated inhibition as indicated by the luciferase reporter assay (Figure 7C). Similarly, disruption of the Nudt21 binding sites in HMGA2 3′-UTR by site-directed mutagenesis (UGUA → TGTA) reduced the inhibitory effects of *let-7* on luciferase activity (Figure 7D).

Based on these results, we generated a possible mechanism showing how Nudt21 regulates HMGA2 expression in hTSCs. Nudt21 binds to the UGUA element upstream of the proximal PAS in HMGA2 3′-UTR, protecting the proximal PAS from cleavage by CPSF. This leads to cleavage and adenylation at the distal site, resulting in the generation of the long 3′-UTR. H_2_O_2_ stimulation increases Nudt21 expression, leading to higher production of the long 3′-UTR. This results in increased *let-7*-mediated silencing and reduced HMGA2 expression.

## DISCUSSION

Since TSCs were first reported in 2007 [3], their role in tendon physiology and pathology has been extensively investigated [8], and their potential therapeutic value as a cell source for tendon repair and regeneration has been demonstrated *in vitro* and *in vivo* [26, 27]. Further, various strategies to maintain the stemness of cultured hTSCs and to optimize their therapeutic effects have been explored [20, 28, 29]. For example, exposure to a pulsed electromagnetic field increases the regenerative potential of hTSCs collected from patients undergoing arthroscopic rotator cuff repair [28]. Rapamycin treatment helps maintain the self-renewal capacity and stemness of hTSCs under elevated oxidative stress by activating autophagy [20]. In a rat model of Achilles tendon injury, rat TSCs (rTSCs) encapsulated in chitosan/β-glycerophosphate/collagen hydrogel significantly enhanced tendon healing compared with rTSCs or hydrogel alone [29]. Understanding the mechanisms governing TSC maintenance and invigoration can cultivate new strategies to optimize TSC-based therapies.

In the present study, we identified HMGA2, a central regulator of stemness and tumorigenicity [10, 11], as a key factor in hTSC stemness maintenance. HMGA2 overexpression prevented H_2_O_2_-induced loss of stemness and differentiation potential in hTSCs, and these protective effects were mediated by activating autophagy. HMGA2 was first linked to autophagy in a 2012 report, where HMGA2 silencing in retinoblastoma cells led to upregulation of damage-regulated autophagy modulator (DRAM) [30], a lysosomal membrane protein required for the induction of autophagy by the p53 pathway [31]. From a study by Yang et al., it was also evident that overexpression of HMGA2 induces autophagy in neurofibromas through its interaction with Musashi-2 promoter region [32]. Later studies have linked HMGA2 to autophagy, where HMGA2 was identified to mediate autophagy by the carcinogen Cr (VI) [33], and HMGA2 overexpression was observed to suppress gefitinib resistance in NSCLC cells by inhibiting autophagy [34]. Additionally, a recent study by Jia et al. showed that HMGA2 overexpression increased proliferation, migration and invasion of glioblastoma cells through activation of Wnt/β-catenin pathway and inhibition of autophagy [35]. These alternating results in various cell types indicate a potential cell type specific relationship of HMGA2 with autophagy. Herein, we report for the first time that HMGA2 upregulates autophagy in hTSCs to protect the cells from oxidative stress-induced impairments. However, there is a need for further investigation to confirm if autophagy serves as a general mechanism by which HMGA2 regulates other types of stem cells.

The regulation of HMGA2 by *let-7* has been well documented [13, 14]. HMGA2 is expressed in embryonic stem cells, but is nearly undetectable in adult somatic cells [36, 37]. This development-dependent drop in HMGA2 expression is caused by a corresponding rise in *let-7* expression [11]. High HMGA2 expression in cancers is commonly associated with under-expression of *let-7* [38]. However, some HMGA2-high cancers have normal levels of *let-7*. In these cancers, the 3′-UTR of the mature HMGA2 mRNA transcript is truncated, allowing HMGA2 to avoid *let-7*-mediated downregulation [17]. Evidence has supported that the truncated 3′-UTRs may arise from chromosomal translocation [13]. In this study, we found that HMGA2 expression in hTSCs depends on APA of its 3′-UTR, which is regulated by Nudt21. Our results showed that H_2_O_2_ stimulation enhances Nudt21 expression and consequent proximal PAS protection, resulting in distal PAS selection and increased *let-7*-mediated silencing. This further leads to reduced HMGA2 expression and loss of hTSC stemness and pluripotency. Interestingly, a study indicated that Nudt21, which is involved in polyadenylation of mRNAs, promotes differentiation of stem cells and cancer cells. Further, it was identified that Nudt21 directly controls polyadenylation of many chromatin regulators during pluripotency and regulates cell fate [39]. However, further studies are warranted to find out whether HMGA2 is regulated by this mechanism in other HMGA2-expressing cell types.

In conclusion, we found that HMGA2 helps maintain stemness and pluripotency of hTSCs under increased oxidative stress. Nudt21-mediated APA of HMGA2 3′-UTR is responsible for H_2_O_2_-induced HMGA2 downregulation and hTSC impairments.

## MATERIALS AND METHODS

### Cell culture and treatment

hTSCs were isolated from supraspinatus tendon of adult patients undergoing arthroscopic rotator cuff repair surgery as previously described [40]. The protocol for human tissue sample collection was approved by the Ethics Committee of Shanghai Sixth People’s Hospital (Shanghai, China). The isolated cells were cultured and expanded in α-minimal essential medium supplemented with penicillin-streptomycin and 10% fetal bovine serum (Thermo Fisher Scientific, Waltham, MA, USA) at 37 °C with 5% CO_2_ following the previous reports [41], and cells in the fourth passage were collected and used in all experiments. To generate increased oxidative stress, cells were treated with 0.25, 0.5 or 0.75 mM H_2_O_2_ for up to 24 h. To evaluate the effects of rapamycin and 3-MA, cells were incubated with 200 nM rapamycin and 2 μM 3-MA, alone or in combination as indicated for 24 h, and then subjected to 24 h H_2_O_2_ treatment.

### Plasmid construction and transfection

The human HMGA2 cDNA sequence (NM_003483.4) was inserted into the GV341 vector (Genechem, Shanghai, China). Two shRNAstargeting NUDT21 (shNudt21-1 and shNudt21-2), a shRNA targeting HMGA2 (shHMGA2) or a scrambled control shRNA were synthesized, inserted into the GV248 vector (Genechem). The targeting sequences were as follows: shNudt21-1, 5′-GCA CCA GGA TAT GGA CCC ATC ATT T-3′; shNudt21-2, 5′-TGA ACC TCC TCA GTA TCC ATA TAT T-3′; shHMGA2, 5′-GCC CAA GGC ACT TTC AAT CTC-3′. For the establishment of stable cell lines, recombinant vectors were packaged to produce lentiviral particles in HEK293T cells according to the manufacturer’s protocol. The hTSCs were infected with lentiviral particles for 72 h (MOI = 50), and then collected for further confirmation. The cells infected with empty vector GV341 or GV248 vector containing scrambled control shRNA served as negative controls,

### Colony formation assay

After treatment, untransfected or transfected hTSCs were seeded in culture dishes at a density of 50 cells/cm^2^ and allowed to grow at 37°C, 5% CO_2_ for 14 days. The cells were washed, fixed in methanol, and stained with crystal violet. Colonies of more than 50 cells were counted using Image-Pro Plus 6.0 software (Bethesda, MD, USA).

### Differentiation assays

The capabilities of hTSCs to differentiate into adipogenic, osteogenic or chondrogenic lineage cells were evaluated as previously described [41, 42]. In brief, cells were cultured in adipogenic, osteogenic or chondrogenic differentiation medium for up to 14 days. The expression of markers for lineage-specific differentiation (PPARγ for adipogenesis, Runx-2 for osteogenesis and Sox-9 for chondrogenesis) was evaluated by qRT-PCR.

### Cell proliferation assay

Cell proliferation was evaluated using the CCK-8 (Dojindo, Kumamoto, Japan) assay. Briefly, untransfected or transfected hTSCs were seeded in 96-well plates at 5 × 10^3^ cells/well and subjected to H_2_O_2_ treatment for 24 h, with or without preincubation with rapamycin or 3-MA as indicated. Cell viability was determined following manufacturer’s instructions. The absorbance at 450 nm representing the number of viable cells was recorded on a Bio-Tek Synergy HT microplate reader.

### Cell cycle analysis

Untransfected and transfected hTSCs were fixed in 70% ethanol, washed with PBS and stained with 50 g/mL propidium iodide (PI; Sigma-Aldrich, St. Louis, Mo, USA) for 30 min in the presence of ribonuclease A (RNaseA; Sigma-Aldrich). The cells were subjected to analysis on a FACSCalibur flow cytometer (Becton Dickinson, San Jose, CA, USA). Cell cycle distribution was calculated using the Cell Quest software (Becton Dickinson).

### Cell senescence analysis

Cell senescence was assessed using the Senescent Cells Staining Kit (Sigma-Aldrich) as previously described [43]. In brief, untransfected and transfected hTSCs were seeded at 1.5 × 10^3^ cells/cm^2^ in 12-well plates and treated with H_2_O_2_ at indicated concentrations for 72 h. The activity of senescence-associated β-galactosidase (SA-β-gal) was detected following manufacturer’s instructions. Images were recorded on an Axiovert S100 light microscope (Carl Zeiss, Oberkochen, Germany) equipped with an AxioCam ICc3 color camera. Cell senescence was calculated as the percentage of β-gal-positive cells based on cell counts from four randomly selected fields.

### Quantitative real-time PCR (qRT-PCR)

Total RNA was extracted using Trizol reagent (Invitrogen, Carlsbad, CA, USA) and reverse transcribed with the RevertAid RT-PCR system (Fermentas, Pittsburgh, PA, USA) following manufacturer’s instructions. The mRNA levels of HMGA2, PPAR-γ, Sox-9, Runx-2 and the short (proximal) and long (distal) HMGA2 3′-UTRs were determined by quantitative PCR on a Mx3000P RT-PCR system (Stratagene, La Jolla, CA, USA) using Maxima SYBR Green qPCR Master Mix (Applied Biosystems, Carlsbad, CA, USA). Primers for HMGA2, Nanog, Nucleostemin, Oct-4, SSEA-4, PPAR-γ, Sox-9 and Runx-2 detection were identical to those published previously [44, 45]. Primers for the detection of the short (proximal) and long (distal) HMGA2 3′-UTRs are listed in Supplementary File 1. Data were normalized to β-actin.

### Western blot analysis

Cells were lysed in RIPA buffer (Thermo Scientific, Waltham, MA, USA) and the protein concentrations were determined using the BCA method (Pierce, Rockford, IL, USA). Samples (20 μg of total protein) were subjected to 12% SDS-PAGE and transferred onto PVDF membranes (Millipore, Bedford, MA, USA). After blocking in 5% nonfat milk for 2 h, the membranes were probed with antibodies towards Nudt21, HMGA2, LC3 and Beclin-1, respectively, at 4°C overnight. All primary antibodies were from Novus Biologicals, Inc. (Littleton, CO, USA). After incubation with horseradish peroxidase-conjugated secondary antibody (Dako, Carpinteria, CA, USA), protein bands were visualized using enhanced chemiluminescent reagents (Beyotime, Shanghai, China). Data were normalized to β-actin.

### Detection of autophagosomes with GFP-LC3 labeling

To detect autophagosome formation, hTSCs were transfected with the GFP-LC3 plasmid (Addgene, Cambridge, MA, USA) for 4 h using Lipofectamine 2000. The transfected cells were seeded at 2 × 10^5^ cells/well in six-well plates and incubated overnight. On the next day, cells were transfected with HMGA2-OE or the empty control vector. After that, the cells were preincubated with rapamycin and 3-MA for 24 h, alone or in combination as indicated, and subjected to 0.5 mM H_2_O_2_ treatment for 24 h. Images were acquired using at least five random vision fields Olympus microscope at 60 × magnification, and the number of GFP-labeled autophagosomes were counted with an Image-Pro Plus 6.0 software.

### 3’ RACE assay

The 3′-end of the HMGA2 mRNA transcripts was analyzed using 3′ RACE. In brief, cDNA was synthesized using an Oligo-dT-adaptor primer with the sequence of 5′-CCA GTG AGC AGA GTG ACG AGG ACT CGA GCT CAA GCT TTT TTT TTT TTT TTT T-3′. The HMGA2 3′-UTR sequences were amplified under standard conditions using primers specific for proximal and distal poly (A) tails (Supplementary File 1) along with the reverse primer 5′-CCA GTG AGC AGA GTG ACG-3′. The PCR products were separated by agarose gel (0.8%) electrophoresis, visualized with SYBR Green, extracted and sequenced.

### Luciferase reporter assays

For luciferase reporter plasmid construction, the full length HMGA2 3′-UTR and a corresponding mutant 3′-UTR with mutations at the Nudt21 binding site (Supplementary File 2) were inserted into the dual-luciferase expression psiCHECK2 vector (Promega) to generate psiCHECK2-HMGA2 and psiCHECK2-HMGA2 MUT plasmids, respectively. The wildtype and mutant 3′-UTR sequences are listed in Supplementary File 2. hTSCs were plated in 24-well plates and cultured to 40–50% confluence. The cells were transfected with 100 ng psiCHECK2-HMGA2 or psiCHECK2-HMGA2 MUT plasmids along with shNudt21-1, shNudt21-2, control shRNA, or *Let-7* as indicated using Lipofectamine 2000 in the absence or presence of 0.5 mM H_2_O_2_. Luciferase activity was determined 48 h after transfection using the Dual Luciferase Assay System (Promega) and normalized to Renilla activity.

### Statistical analysis

All results are presented as the mean ± SD (standard deviation). Data were analyzed using GraphPad Prism 5 (GraphPad Software Inc., La Jolla, CA, USA). Differences between treatment groups were interpreted using one-way analysis of variance. Single and multiple comparisons were conducted with Student’s *t* test and Tukey’s HSD post hoc test, respectively. Differences with p < 0.05 were considered statistically significant.

## Supplementary Material

Supplementary Figure 1

Supplementary File 1

Supplementary File 2
