# Supplementary material for: Nudt21-mediated alternative polyadenylation of HMGA2 3′-UTR impairs stemness of human tendon stem cell
Source: Aging (Albany NY). 2020 Sep 26;12(18):18436–52. doi: 10.18632/aging.103771 (PMC7585117; doi:10.18632/aging.103771)
Supplement: Supplementary Figure 1 [file aging-12-103771-s002..pdf]

SUPPLEMENTARY FIGURE

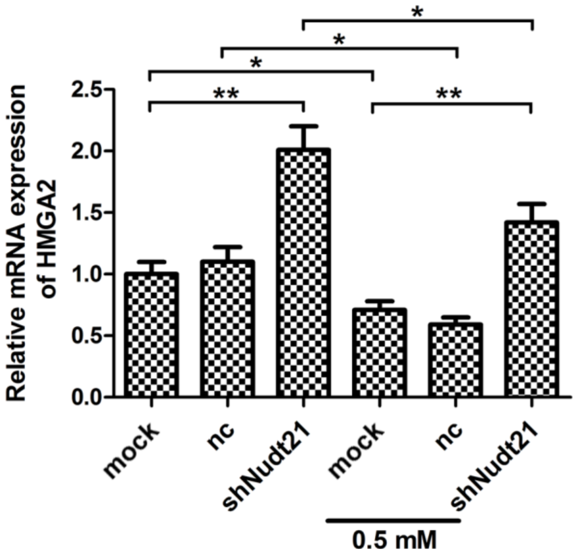

Supplementary Figure 1. Relative gene expression of HMGA2. hTSCs transfected with shNudt21-2 or a scrambled control shRNA (nc) were cultured in the presence or absence of 0.5 mM H_2O_2 for 24 h. Untransfected cells (mock) were included for comparison. The mRNA levels of HMGA2 were detected by RT-PCR.
