# Supplementary material for: Nudt21-mediated alternative polyadenylation of HMGA2 3′-UTR impairs stemness of human tendon stem cell
Source: Aging (Albany NY). 2020 Sep 26;12(18):18436–52. doi: 10.18632/aging.103771 (PMC7585117; doi:10.18632/aging.103771)
Supplement: Supplementary File 1 [file aging-12-103771-s003..pdf]

SUPPLEMENTARY FILE

Supplementary File 1. Primer sequences for 3’-UTR analysis.

**qRT-PCR**	for figure 7a			
	HMGA2	proximal	forward	AGGAAGCAGCAGCAAGAA
			reverse	CTAAACCTGGGACTGTGAA
		distal	forward	ACCCAGGGGAAGACCCAAA
			reverse	CCTCTTGGCCGTTTTTCTCCA
**3'-RACE**	for figure 7b			
				forward
	HMGA2-distal			AGCTGGAAGGAAGTTGTTGAA
	HMGA2-proximal			TGTACCATCAAAGCTTCAGAAGA
