# Supplementary material for: Nudt21-mediated alternative polyadenylation of HMGA2 3′-UTR impairs stemness of human tendon stem cell
Source: Aging (Albany NY). 2020 Sep 26;12(18):18436–52. doi: 10.18632/aging.103771 (PMC7585117; doi:10.18632/aging.103771)
Supplement: Supplementary File 2 [file aging-12-103771-s001..docx]

**Supplementary file 2 Let-7 target HMGA2 3'UTR:**

GGGGCGCCAACGTTCGATTTCTACCTCAGCAGCAGTTGGATCTTTTGAAGGGAGAAGACACTGCAGTGACCACTTATTCTGTATTGCCATGGTCTTTCCACTTTCATCTGGGGTGGGGTGGGGTGGGGTGGGGGAGGGGGGGGTGGGGTGGGGAGAAATCACATAACCTTAAAAAGGACTATATTAATCACCTTCTTTGTAATCCCTTCACAGTCCCAGGTTTAGTGAAAAACTGCTGTAAACACAGGGGACACAGCTTAACAATGCAACTTTTAATTACTGTTTTCTTTTTTCTTAACCTACTAATAGTTTGTTGATCTGATAAGCAAGAGTGGGCGGGTGAGAAAAACCGAATTGGGTTTAGTCAATCACTGCACTGCATGCAAACAAGAAACGTGTCACACTTGTGACGTCGGGCATTCATATAGGAAGAACGCGGTGTGTAACACTGTGTACACCTCAAATACCACCCCAACCCACTCCCTGTAGTGAATCCTCTGTTTAGAACACCAAAGATAAGGACTAGATACTACTTTCTCTTTTTCGTATAATCTTGTAGACACTTACTTGATGATTTTTAACTTTTTATTTCTAAATGAGACGAAATGCTGATGTATCCTTTCATTCAGCTAACAAACTAGAAAAGGTTATGTTCATTTTTCAAAAAGGGAAGTAAGCAAACAAATATTGCCAACTCTTCTATTTATGGATATCACACATATCAGCAGGAGT*AATAAA*TTTACTCACAGCACTTGTTTTCAGGACAACACTTCATTTTCAGGAAATCTACTTCCTACAGAGCCAAAATGCCATTTAGCAATAAATAACACTTGTCAGCCTCAGAGCATTTAAGGAAACTAGACAAGTAAAATTATCCTCTTTGTAATTTAATGAAAAGGTACAACAGAATAATGCATGATGAACTCACCTAATTATGAGGTGGGAGGAGCGAAATCTAAATTTCTTTTGCTATAGTTATACATCAATTTAAAAAGCAAAAAAAAAAAAGGGGGGGGCAATCTCTCTCTGTGTCTTTCTCTCTCTCTCTTCCTCTCCCTCTCTCTTTTCATTGTGTATCAGTTTCCATGAAAGACCTGAATACCACTTACCTCAAATTAAGCATATGTGTTACTTCAAGTAATACGTTTTGACATAAGATGGTTGACCAAGGTGCTTTTCTTCGGCTTGAGTTCACCATCTCTTCATTCAAACTGCACTTTTAGCCAGAGATGCAATATATCCCCACTACTCAATACTACCTCTGAATGTTACAACGAATTTACAGTCTAGTACTTATTACATGCTGCTATACACAAGCAATGCAAGAAAAAAACTTACTGGGTAGGTGATTCTAATCATCTGCAGTTCTTTTTGTACACTTAATTACAGTTAAAGAAGCAATCTCCTTACTGTGTTTCAGCATGACTATGTATTTTTCTATGTTTTTTTAATTAAAAATTTTTAAAATACTTGTTTCAGCTTCTCTGCTAGATTTCTACATTAACTTGAAAATTTTTTAACCAAGTCGCTCCTAGGTTCTTAAGGATAATTTTCCTCAATCACACTACACATCACACAAGATTTGACTGTAATATTTAAATATTACCCTCCAAGTCTGTACCTCAAATGAATTCTTTAAGGAGATGGACTAATTGACTTGCAAAGACCTACCTCCAGACTTCAAAAGGAATGAACTTGTTACTTGCAGCATTCATTTGTTTTTTCAATGTTTGAAATAGTTCAAACTGCAGCTAACCCTAGTCAAAACTATTTTTGTAAAAGACATTTGATAGAAAGGAACACGTTTTTACATACTTTTGCAAAATAAGTAAATAATAAATAAAATAAAAGCCAACCTTCAAAGAAACTTGAAGCTTTGTAGGTGAGATGCAACAAGCCCTGCTTTTGCATAATGCAATCAAAAATATGTGTTTTTAAGATTAGTTGAATATAAGAAAATGCTTGACAAATATTTTCATGTATTTTACACAAATGTGATTTTTGTAATATGTCTCAACCAGATTTATTTTAAACGCTTCTTATGTAGAGTTTTTATGCCTTTCTCTCCTAGTGAGTGTGCTGACTTTTTAACATGGTATTATCAACTGGGCCAGGAGGTAGTTTCTCATGACGGCTTTTGTCAGTATGGCTTTTAGTACTGAAGCCAAATGAAACTCAAAACCATCTCTCTTCCAGCTGCTTCAGGGAGGTAGTTTCAAAGGCCACATACCTCTCTGAGACTGGCAGATCGCTCACTGTTGTGAATCACCAAAGGAGCTATGGAGAGAATTAAAACTCAACATTACTGTTAACTGTGCGTTAAATAAGCAAATAAACAGTGGCTCATAAAAATAAAAGTCGCATTCCATATCTTTGGATGGGCCTTTTAGAAACCTCATTGGCCAGCTCATAAAATGGAAGCAATTGCTCATGTTGGCCAAACATGGTGCACCGAGTGATTTCCATCTCTGGTAAAGTTACACTTTTATTTCCTGTATGTTGTACAATCAAAACACACTACTACCTCTTAAGTCCCAGTATACCTCATTTTTCATACTGAAAAAAAAAGCTTGTGGCCAATGGAACAGTAAGAACATCATAAAATTTTTATATATATAGTTTATTTTTGTGGGAGATAAATTTTATAGGACTGTTCTTTGCTGTTGTTGGTCGCAGCTACATAAGACTGGACATTTAACTTTTCTACCATTTCTGCAAGTTAGGTATGTTTGCAGGAGAAAAGTATCAAGACGTTTAACTGCAGTTGACTTTCTCCCTGTTCCTTTGAGTGTCTTCTAACTTTATTCTTTGTTCTTTATGTAGAATTGCTGTCTATGATTGTACTTTGAATCGCTTGCTTGTTGAAAATATTTCTCTAGTGTATTATCACTGTCTGTTCTGCACAATAAACATAACAGCCTCTGTGATCCCCATGTGTTTTGATTCCTGCTCTTTGTTACAGTTCCATTAAATGAGTAATAAAGTTTGGTCAAAACAGAAAAAAAAAAA

**HMGA2 mutant**

GGGGCGCCAACGTTCGATTTCTACCTCAGCAGCAGTTGGATCTTTTGAAGGGAGAAGACACTGCAGTGACCACTTATTCTGTATTGCCATGGTCTTTCCACTTTCATCTGGGGTGGGGTGGGGTGGGGTGGGGGAGGGGGGGGTGGGGTGGGGAGAAATCACATAACCTTAAAAAGGACTATATTAATCACCTTCTTTGTAATCCCTTCACAGTCCCAGGTTTAGTGAAAAACTGCTGTAAACACAGGGGACACAGCTTAACAATGCAACTTTTAATTACTGTTTTCTTTTTTCTTAACCTACTAATAGTTTGTTGATCTGATAAGCAAGAGTGGGCGGGTGAGAAAAACCGAATTGGGTTTAGTCAATCACTGCACTGCATGCAAACAAGAAACGTGTCACACTTGTGACGTCGGGCATTCATATAGGAAGAACGCGGTGTGTAACACTGTGTACACCTCAAATACCACCCCAACCCACTCCCTGTAGTGAATCCTCTGTTTAGAACACCAAAGATAAGGACTAGATACTACTTTCTCTTTTTCGTATAATCTTGTAGACACTTACTTGATGATTTTTAACTTTTTATTTCTAAATGAGACGAAATGCTGACAGCTCCTTTCATTCAGCTAACAAACTAGAAAAGGTTATGTTCATTTTTCAAAAAGGGAAGTAAGCAAACAAATATTGCCAACTCTTCTATTTATGGATATCACACATATCAGCAGGAGT*AATAAA*TTTACTCACAGCACTTGTTTTCAGGACAACACTTCATTTTCAGGAAATCTACTTCCTACAGAGCCAAAATGCCATTTAGCAATAAATAACACTTGTCAGCCTCAGAGCATTTAAGGAAACTAGACAAGTAAAATTATCCTCTTCAGCATTTAATGAAAAGGTACAACAGAATAATGCATGATGAACTCACCTAATTATGAGGTGGGAGGAGCGAAATCTAAATTTCTTTTGCTATAGTTATACATCAATTTAAAAAGCAAAAAAAAAAAAGGGGGGGGCAATCTCTCTCTGTGTCTTTCTCTCTCTCTCTTCCTCTCCCTCTCTCTTTTCATTGTGTATCAGTTTCCATGAAAGACCTGAATACCACTTACCTCAAATTAAGCATATGTGTTACTTCAAGTAATACGTTTTGACATAAGATGGTTGACCAAGGTGCTTTTCTTCGGCTTGAGTTCACCATCTCTTCATTCAAACTGCACTTTTAGCCAGAGATGCAATATATCCCCACTACTCAATACTACCTCTGAATGTTACAACGAATTTACAGTCTAGTACTTATTACATGCTGCTATACACAAGCAATGCAAGAAAAAAACTTACTGGGTAGGTGATTCTAATCATCTGCAGTTCTTTTTGTACACTTAATTACAGTTAAAGAAGCAATCTCCTTACTGTGTTTCAGCATGACTATGTATTTTTCTATGTTTTTTTAATTAAAAATTTTTAAAATACTTGTTTCAGCTTCTCTGCTAGATTTCTACATTAACTTGAAAATTTTTTAACCAAGTCGCTCCTAGGTTCTTAAGGATAATTTTCCTCAATCACACTACACATCACACAAGATTTGACTGTAATATTTAAATATTACCCTCCAAGTCTGTACCTCAAATGAATTCTTTAAGGAGATGGACTAATTGACTTGCAAAGACCTACCTCCAGACTTCAAAAGGAATGAACTTGTTACTTGCAGCATTCATTTGTTTTTTCAATGTTTGAAATAGTTCAAACTGCAGCTAACCCTAGTCAAAACTATTTTTGTAAAAGACATTTGATAGAAAGGAACACGTTTTTACATACTTTTGCAAAATAAGTAAATAATAAATAAAATAAAAGCCAACCTTCAAAGAAACTTGAAGCTTTGTAGGTGAGATGCAACAAGCCCTGCTTTTGCATAATGCAATCAAAAATATGTGTTTTTAAGATTAGTTGAATATAAGAAAATGCTTGACAAATATTTTCATGTATTTTACACAAATGTGATTTTTGTAATATGTCTCAACCAGATTTATTTTAAACGCTTCTTATGTAGAGTTTTTATGCCTTTCTCTCCTAGTGAGTGTGCTGACTTTTTAACATGGTATTATCAACTGGGCCAGGAGGTAGTTTCTCATGACGGCTTTTGTCAGTATGGCTTTTAGTACTGAAGCCAAATGAAACTCAAAACCATCTCTCTTCCAGCTGCTTCAGGGAGGTAGTTTCAAAGGCCACATACCTCTCTGAGACTGGCAGATCGCTCACTGTTGTGAATCACCAAAGGAGCTATGGAGAGAATTAAAACTCAACATTACTGTTAACTGTGCGTTAAATAAGCAAATAAACAGTGGCTCATAAAAATAAAAGTCGCATTCCATATCTTTGGATGGGCCTTTTAGAAACCTCATTGGCCAGCTCATAAAATGGAAGCAATTGCTCATGTTGGCCAAACATGGTGCACCGAGTGATTTCCATCTCTGGTAAAGTTACACTTTTATTTCCTGTATGTTGTACAATCAAAACACACTACTACCTCTTAAGTCCCAGTATACCTCATTTTTCATACTGAAAAAAAAAGCTTGTGGCCAATGGAACAGTAAGAACATCATAAAATTTTTATATATATAGTTTATTTTTGTGGGAGATAAATTTTATAGGACTGTTCTTTGCTGTTGTTGGTCGCAGCTACATAAGACTGGACATTTAACTTTTCTACCATTTCTGCAAGTTAGGTATGTTTGCAGGAGAAAAGTATCAAGACGTTTAACTGCAGTTGACTTTCTCCCTGTTCCTTTGAGTGTCTTCTAACTTTATTCTTTGTTCTTTATGTAGAATTGCTGTCTATGATTGTACTTTGAATCGCTTGCTTGTTGAAAATATTTCTCTAGTGTATTATCACTGTCTGTTCTGCACAATAAACATAACAGCCTCTGTGATCCCCATGTGTTTTGATTCCTGCTCTTTGTTACAGTTCCATTAAATGAGTAATAAAGTTTGGTCAAAACAGAAAAAAAAAAA

Poly(A) tail signal sequence (PAS: AATAAA) and Nudt21 recognition sites (TGTA) are underlined. The *Let-7* binding sites are highlighted in yellow.
